# Modern pea (*Lathyrus oleraceus*) cultivars exhibit significantly lower genetic diversity relative to landraces, garden cultivars and genebank accessions

**DOI:** 10.1371/journal.pone.0357113

**Published:** 2026-10-21

**Authors:** Jens Axel Knuhtsen, Conny Bruun Asmussen Lange, Dorte Bodin Dresbøll, Kristian Holst Laursen, Karen Rysbjerg Jensen, Søren Kjærsgaard Rasmussen

**Affiliations:** Department of Plant and Environmental Sciences, University of Copenhagen, Copenhagen, Denmark; Julius Kuhn-Institut, GERMANY

## Abstract

Pea (*Lathyrus oleraceus* Lam.), formerly (*Pisum sativum* L.) is an important grain legume, cultivated worldwide for both food and feed purposes. Gene banks safeguard and maintain the genetic resources of the species. The Nordic Genetic Resource Center (NordGen) manages approximately 4,000 pea accessions. In gene banks, the proportion of genotyped accessions is low. This low number limits available information on accessions, increases the risk of duplicates, and reduces the resource efficiency and applicability of gene bank material. Our study included 304 accessions, comprising 18 modern field pea cultivars and 22 garden pea cultivars. All accessions were genotyped using Diversity Arrays Technology sequencing genotyping. This yielded 30,791 single nucleotide polymorphic markers after quality control. Genetic population structure was analyzed and visualized using neighbor-joining trees and Principal Coordinate Analysis plots. The genetic diversity in the population was high. All modern cultivars clustered together in the Principal Coordinate Analysis plots and were resolved as a monophyletic group with high bootstrap support in the neighbor-joining tree. The garden peas were dispersed and intermixed with historic landraces of European origin. Nepalese and Indian landraces, in conjunction with Abyssinian pea accessions, were resolved as a sister group to the rest of the population. Our results demonstrated significant genetic homogeneity in modern cultivars relative to the subset of accessions analyzed. The population structures found in our analyses added to the limited number of publications on genetic diversity, based on large numbers of genetic markers in pea collections. The results supported the often said, but rarely proven hypothesis, that modern cultivars display reduced genetic diversity in agricultural crops.

## Introduction

### Pea – Introduction, taxonomy and cultivation history

Pea (*Lathyrus oleraceus* Lam*.*, syn. *Pisum sativum* L.) is a diploid species (2n = 14), consistent with all members of the genus *Lathyrus* [1] in the pea family. Based on molecular data, it has been shown that the genus *Pisum* is included in *Lathyrus* [1–3], leading to a consequential renaming of the former *Pisum* species. Despite this, most literature, breeding companies and gene banks still adhere to the *Pisum* nomenclature. For this reason, the present manuscript will use the well-known *Pisum* synonyms to ease the comparison with existing literature. The genome sequence of the pea is 3.92 giga bases [4], and it is typically classified as a self-pollinated annual plant [5]. Pea cultivation began in the Near East, and the species was cultivated at least as early as 7,000–9,000 years ago [6]. Various forms of wild peas are recorded in areas around the Mediterranean, as well as in southern Europe and northern Africa [1]. Currently, peas are cultivated globally and have been adapted to temperate climates, including those found in Denmark [7]. Archaeological evidence shows that peas have been cultivated in Denmark since the Pre-Roman Iron Age, over 2,000 years ago [8]. Today, pea is considered the most important protein crop in the Nordic countries and is a high-potential crop utilized as food, feed, and forage [9].

### Genetic population structure studies

Many studies have examined genetic diversity in pea collections. These studies include garden peas, forage peas, landraces, and historical collections from various regions and countries including the Czech Republic, Turkey, Tunisia, Anatolia, Russia, Australia, and India. The pea populations were analyzed using simple sequence repeats (SSR) [10–13]. The polymorphic information content (PIC) for a range of SSR markers ranged from a few to several hundred. The PIC values varied from slightly informative (<0.25), reasonably informative (0.25 to 0.5), to highly informative (>0.5) [10,14,15]. A core set of SSR markers can be used as a low-cost method to assess populations and select traits. The DArTseq™ technology is a state-of-the-art method to discover genetic markers in a wide range of species [16]. Diversity Arrays Technology (DArT) sequencing can be used for developing Single Nucleotide Polymorphism (SNP) markers for genetic diversity analysis and has been applied to pea collections [17,18]. Like SSR markers, DArT does not necessitate prior knowledge of genomic sequences, although such information is available for peas [4]. The genome coverage of DArT sequencing is high [16]. A study focused on a dedicated breeding population of 188 wrinkled vining pea accessions resulted in the development of 6,902 SNP markers using the DArTseq technology [18]. The chromosomal distribution of these SNP markers ranged from 401 on chromosome 3LGS to 1,062 on chromosome 5LGS. PIC values ranged from 0.006 to 0.38, averaging 0.27. The genome-wide physical distance at which linkage disequilibrium (LD) decays to half of its initial value is estimated to be 6.93 megabase pairs (Mbp), with observed variation across different chromosomes. A global pea study with 265 accessions from five gene banks produced 6,966 SNP markers [17]. Most SNP markers in this diverse pea collection were mapped to chromosome 5 (1,422), while the fewest were mapped to chromosome 1 (734). The PIC values of the SNP markers ranged from 0.0 to 0.5, with 25% of them exhibiting PIC values greater than 0.4, which are considered informative. Linkage disequilibrium analyses showed that the genetic distance between markers was 34 Kb in that pea collection. In a study of 108 recombinant inbred lines, 6,447 markers had PIC values ranging from 0.34 to 0.51, averaging 0.44 [19]. A study involving 325 accessions of pea, encompassing seven subspecies, generated a total of 11,508 DArT markers. In that study, each chromosome had an average of 1,644 markers, with chromosome 2 having 1,068 and chromosome 5 having 1,993. The PIC values ranged between 0.013 and 0.594, with an average of 0.267, while the LD50 ranged from 0.48 to 1.38 Mbp [20]. The PIC values obtained in that study indicated that DArT-derived SNP markers are informative.

### Pea in a Scandinavian perspective

The official variety trial of field pea in Denmark tested an average of 26 cultivars each year from 1995–2000. From 2001–2006, 12 cultivars were tested per year, followed by eight years without new introductions. Since 2015, the number of annual trials of field pea have grown from two or three to seven cultivars in 2023, demonstrating increased efforts in plant breeding [21]. To our knowledge, no studies have investigated the genetic structure of the Scandinavian germplasm using the DArTseq genotyping platform. To explore existing genetic resources adapted to the temperate climate of Southern Scandinavia, we analyzed 330 individuals from 304 accessions. Most of these accessions originate from the collection of The Nordic Genetic Resource Center (NordGen). The accessions were selected based on the indications of a high protein content. In the analysis, we included 18 modern field pea cultivars, 22 garden peas sold in Northern Europe and several landraces of mostly Danish and Swedish origin. This study aims to enhance the understanding of the genetic population structure of pea accessions primarily of Scandinavian origin. In addition, comparing these Scandinavian gene bank accessions with modern field pea cultivars currently available to farmers.

### Research questions and outlook

The research questions we pursued were: 1) What is the population structure of the selected pea accessions? 2) How do modern cultivars, garden peas, and landraces cluster relative to each other? 3) What is the level of diversity of all the pea accessions, and within modern cultivars, garden peas, and landraces specifically? These research questions are based on the following hypotheses: 1) The modern cultivars, landraces, and garden peas cluster in three separate groups. 2) Landraces position as a genetic middle point between the modern cultivars and the garden peas. 3) The diversity within the modern cultivars is significantly smaller than that of garden peas and landraces. The results are expected to provide insight into the present level of genetic diversity in modern pea cultivars used in Scandinavian cropping systems, and to investigate the genetic resources that hold the potential for increasing genetic diversity in future breeding.

## Materials and methods

### The population

The population investigated totaled 330 individuals originating from 304 accessions. To create each individual, a single seed from a NordGen accession was subjected to a ‘Single Seed Descent’ procedure. The single seed was grown and harvested, and this seed lot was then considered the individual. In some cases, multiple seeds were grown from the same accession. In this way we created multiple individuals per accession. Accession selection was primarily based on indications of high protein content, and a northern European cultivation history. 241 accessions were selected from the *Pisum sativum* collection of NordGen (www.nordgen.org). Additionally, 18 commercially available pea cultivars marketed as field peas, and 22 pea cultivars purchased as garden peas were included. Finally, 23 accessions were acquired from the University of Copenhagen, containing historic cultivars from the first half of the 1900s (S1 File*)*. Preliminary SNP quality and accession pruning led to the final population containing 330 individuals representing 304 accessions, and included landraces, breeding lines, garden peas, and improved cultivars. The population contained various pea types, including field pea, garden pea, climbing types, dwarf types, snap peas and marrowfat peas. Thus, the population represented a large variation of morphological diversity and end-use purposes. The commercial cultivars were bought from web shops marketing garden peas or supplied by agricultural research institutions. The commercial field pea cultivars were selected to represent the field peas included in the National Variety Tests in Denmark in the period of 2020–2024. This included peas from European breeding companies, including Nordic Seed (Denmark), Sejet Plant Breeding (Denmark), Lantmännen (Sweden), KWS (Germany), and Unisigma (France). The garden peas were selected to reflect the diversity present in European garden peas. Where data were available, the breeding company, region of origin, year of release or first records of national cultivar testing, was included (S1 File)*.* Each NordGen accession was queried in the database available at https://www.nordic-baltic-genebanks.org/gringlobal/search and relevant data was extracted (S1 File*)*. The resulting population represented accessions from several countries and breeding companies and covered more than 100 years of pea cultivation history.

### Field setup and phenotypical data

To record phenotypes, a subset of 211 out of the 304 accessions were cultivated in-field. The remainder of the accessions did not produce enough seeds for single row field evaluation during the ‘Single Seed Descent’ procedure. The single row field evaluation was established at the field research station of University of Copenhagen, Denmark, Taastrup (55°40’08.7“N 12°18’16.5”E) on the 27^th^ of April 2023 on a loamy sandy soil. Of the 211 accessions, two failed to germinate in the field. For each accession, ten seeds were sown per meter at a depth of five cm, with one meter between rows. Each row was 15 m long and thus contained 15 accessions. Before germination, a trellis system was installed for plant support. Weeds were excluded using water-permeable polypropylene weed mats. The setup is presented in the (S2 File). Each plant was supplied with ten grams of an organic fertilizer based on chicken manure, thus supplying 0.4 g of nitrogen, 0.28 g of potassium, and 0.08 g of phosphorus per plant. This fertilizer was applied as topdressing three weeks after germination. During the season, weeds were removed, and plant attachment to the trellis was checked. The plants were manually irrigated twice during dry periods but were otherwise rainfed. Each accession was hand harvested at maturity, and pods were hand-threshed after a few weeks of drying in paper bags. No pesticides or rhizobia inoculum were applied. Because it was a relatively dry year, no severe disease outbreaks were recorded. An aphid (*Aphididae*; multiple species) infestation occurred but was diminished after a few weeks by a sudden increase in ladybug (*Coccinella septempunctata*) numbers. Thousand-seed-weight was measured using a Seed Counter (Contador, Pfeuffer GmbH, Kitzingen, Germany). Genetically stable morphological traits (leaf morphology and flower color) were recorded either in the field or when growing plants in pots for DNA extraction. Leaf morphology determination followed the description and images presented in Tayeh et al. (2024) [22]. This included three variations: 1) “tendrilless”, which displayed no tendrils but rather had 6 pairs of leaflets, 2) “wild-type”, in which the 3 terminal leaflet pairs were replaced with tendrils, and 3) “*afila*”, in which all leaflets were converted into tendrils. The color of the flower was recorded as white, purple, or white-and-pink. In the white-and-pink case, the banner was white, while the keel and/or the wings displayed some variant of pink and purple coloration. Seed morphology (surface type) was recorded on dry seeds and determined as either wrinkled or smooth.

The single row field evaluation yielded the morphological traits “thousand-seed-weight”, “leaf morphology”, “flower color” and “seed surface type”. The latter three traits are qualitative traits stable across environments. As for the quantitative trait thousand-seed-weight, only one biological replicate, i.e., plot was grown per genotype. This means that care should be taken when interpreting the thousand-seed-weight of individual accessions.

### DNA extraction and DArT genotyping

The CTAB method [23] was followed to extract total genomic DNA from silica gel-dried leaf material from fully developed leaves on young plants, cultivated in pots in the greenhouse. The concentration and purity of the total genomic DNA samples were measured using a deNovix spectrophotometer (Wilmington, Delaware, USA). Samples with concentrations below 25 ng/ml were re-extracted. Purity was measured as the absorbance at 260 and 280 nm (A260/280). Samples with a ratio of A260/280 between 1.6 and 2.0 (1.8 ± 0.2) were considered pure. Non-pure samples were re-extracted. After dissolving the DNA pellet in a 50 μL 1 × TE buffer, the quality of the DNA was assessed on an agarose gel (VWR PerfectBlue, VWR SMART5, VWR International) and using spectrophotometry on a Uvidoc (Buch & Holm, Herlev, Denmark). Total genomic DNA was diluted to a concentration of 50–100 ng/µL, and 30 µL of each sample was aliquoted into a 96-well DNA plate following the guidelines provided by Diversity Arrays Technology (https://help.diversityarrays.com/docs/genomicdna-gdna). The plate was shipped to Diversity Arrays Technology, Canberra, Australia (https://www.diversityarrays.com/) for genotyping by the DArTSeq-approach. DArT sequencing and DNA quality control followed the methods described by Diversity Arrays Technology [16].

### Data preparation, quality control, and accession filtering

Genotype datasets were received from DArT. To reduce complexity of the analysis, the silico-DArT dataset was not included in the analysis. Genetic data was managed in R version 4.4.2 [24]. The  .ped and  .map-file generation, as well as quality control, was performed using PLINK v. 1.9 [25]. The MAF threshold was set to 0.05, and markers missing in more than 20% of the accessions were removed. Following quality control, the chromosomal density distribution of the resulting SNPs was visualized using the online tool “SNP density” on the platform “SRplot” [26]. The SNP PIC values were summarized from the “PICSnp” column supplied in the original dataset from DArT. The PIC data extraction was only run on the SNPs passing quality control and thus included in the main analyses. Preliminary screening revealed that the population included accessions that appeared to originate from two mutant populations generated by induced mutation on single cultivars. Accessions from those populations shared >90% of SNPs after quality control. In GeNBIS, accessions from the mutant populations either had cultivars ‘Weitor’ or ‘Parvus’ listed as progenitor, as well as information on which mutagen the accession was generated with. This led to the conclusion that those accessions should be removed from the population, as they would otherwise lead to sampling bias. Six accessions with ‘Parvus’ pedigree and 26 with ‘Weitor’ pedigree were removed before the main analysis. The accessions NGB 101304 and NGB 101997 were selected to investigate the within-accession SNP variation. From these accessions, 10 seeds were sown, DNA extracted, and DArTseq genotyped as individuals. This was done to check the genetic diversity within single accessions. To avoid overrepresentation of individual accessions, only one individual from each accession was included in the population structure analyses. After removing the mutant population and the accession replicates, the final analysis was conducted on 279 individual genotypes.

### Population structure

Population structure was inferred using STRUCTURE version 2.3.4 [27].

Simulations were repeated 10 times with K ranging from 1–15, 10,000 burn-in, and 20,000 MCMC iterations. CLUMPAK (beta-version accessed 15.11.24) [28] was used on the output to determine the optimal K [29]. Data for Principal Coordinate Analysis (PCoA) plots were prepared using PLINK v. 1.9 to generate eigenvectors [25]. The number of principal components was set to be equal to the number of accessions. The eigenvectors were then visualized as a PCoA plot using the R-package ggplot2 v. 3.5.1 [30]. Subpopulation color visualized in the NJ-tree (neighbor-joining) and in the PCoA plot were derived from the STRUCTURE output. Accessions were considered as belonging to a subpopulation if they shared at least 80% identity with that subpopulation. The Best K by the Evanno method-plot was generated via ggplot2 from the values outputted by the CLUMPAK ‘Best K’-pipeline.

### Neighbor joining tree

The dataset was manually converted to  .fasta-format and uploaded to MEGA11 [31] for NJ-tree construction. In MEGA, the genotypes were read as non-coding nucleotide sequences. 2000 Boot strap replicates were generated [32]. The substitution model was the Kimura 2-parameter model [33]. All other parameters were set to default. The *Pisum sativum* subsp. *abyssinicum*-subgroup was chosen as an outgroup for rooting the tree. This choice was justified based on molecular evidence that *Pisum sativum* subsp. *abyssinicum* was domesticated independently of other *Pisum sativum* subspecies [34]. For this reason, it was considered a distinct accession group outside the rest of the population. The tree was exported as a Newick file, and the visual representation was performed using the ggTree-package v. 3.14.0 [35], following the excellent manual [36].

### Statistical analysis

Basic statistical procedures were performed in R version 4.4.2 [24]. Genetic diversity parameters and genetic differentiation were estimated on the post-quality control SNP dataset using the R package ‘Hierfstat’ v. 0.5.11 [37]. These analyses provided estimates for observed heterozygosity (Ho), expected heterozygosity (He), and Weir and Cockerhams pairwise fixation index (Fst). Inbreeding coefficients (Fis) were calculated as Fis = 1 – (Ho/ He). Other statistical procedures included the calculation of averages, means, and standard deviations, in relation to thousand-seed-weight measurements, as well as calculating the percentage of shared SNPs between accession individuals. Associations between subpopulations and categorical phenotypes were investigated using chi-squared (χ^2^) tests of independence and Pearson residuals. The subpopulation assignments were derived from the STRUCTURE analysis. The phenotypes were the categorical phenotypes visualized on the NJ-tree (flower color, pea surface type and leaf morphology). First the data was checked for assumptions for the χ^2^ test. Each accession was treated as an independent observation, and no accession was duplicated in the association analysis. To meet the assumption of adequate sample sizes, the expected cell counts of the χ^2^ approximation were considered. Expected cell counts <1 and with more than 20% of cells with expected counts <5 was determined not to meet the assumptions. To meet them the following phenotypic data categories had to be collapsed: Flower colour categories were collapsed into “white” or “pigmented”. The “pigmented” category consisted of flower colors “pink” n = 5, “white-and-purple” n = 4 and “purple” n = 50. The “white” category was n = 174. Leaf morphology did not meet the adequate sample sizes assumption either. “*afila*” (n = 31) and “wild-type” (n = 190) were kept while including “tendril-less” (n = 7) resulted in expected counts <1. Therefore the 7 “tendril-less” records were excluded from the analysis leaving only the “wild-type” and “*afila*” leaf morphologies in the analysis. Following these modifications, data now met the assumptions for the χ^2^ test (expected cell counts >1 and >20% of cells with expected counts >5). After these modifications, assumptions were tested again and found to be adequate. The categorical phenotype data were analyzed individually per trait. Due to the data modifications described, sample sizes varied among traits. No imputation was performed to remove missing values. A contingency table was constructed individually for each trait. Only complete cases were included – meaning only accessions with recorded phenotypes and subpopulation assignment. The χ^2^ p-values were extracted to evaluate the overall independence between phenotypes and subpopulations. Pearson residuals were extracted to quantify specific phenotype-subpopulation deviations from independence. χ^2^ and Pearson residuals were derived using r-package “dplyr” v. 1.1.4 [38].

## Results

### Distribution of DArTseq-based SNP markers

It was possible to identify 30,791 SNPs using DArTseq technology, and quality filtering resulted in 8,062 SNP loci in the working pea collection of pea accessions. Most of the SNPs (6,790) could be mapped to the seven pea chromosomes (Fig 1), leaving 1,272 SNPs (16%) unassigned. The number of SNPs per chromosome ranged from 756 to 1,356, and they were well distributed along the chromosomes, making them suitable for future genetic studies. Marker density ranged from 1.7 SNPs per Mb to 2.2 SNPs per Mb for the seven chromosomes. The PIC values of our SNPs after quality control were evenly distributed across a range from 0 to 0.5, with 49% SNPs having a PIC > 0.25 and 51% between 0 and 0.25. Given the distribution of the PIC values, both poorly informative and highly informative SNPs were available for the analysis (Table 1).

**Table 1:** PIC value range for the 8,062 DArT-seq-based SNPs used in both the STRUCTURE analysis, and the NJ-tree construction.

PIC value range	Percentage of SNPs in range
Range 0.0–0.10	22
Range 0.10–0.25	29
Range 0.25–0.40	22
Range 0.40–0.50	27

**Fig 1:**
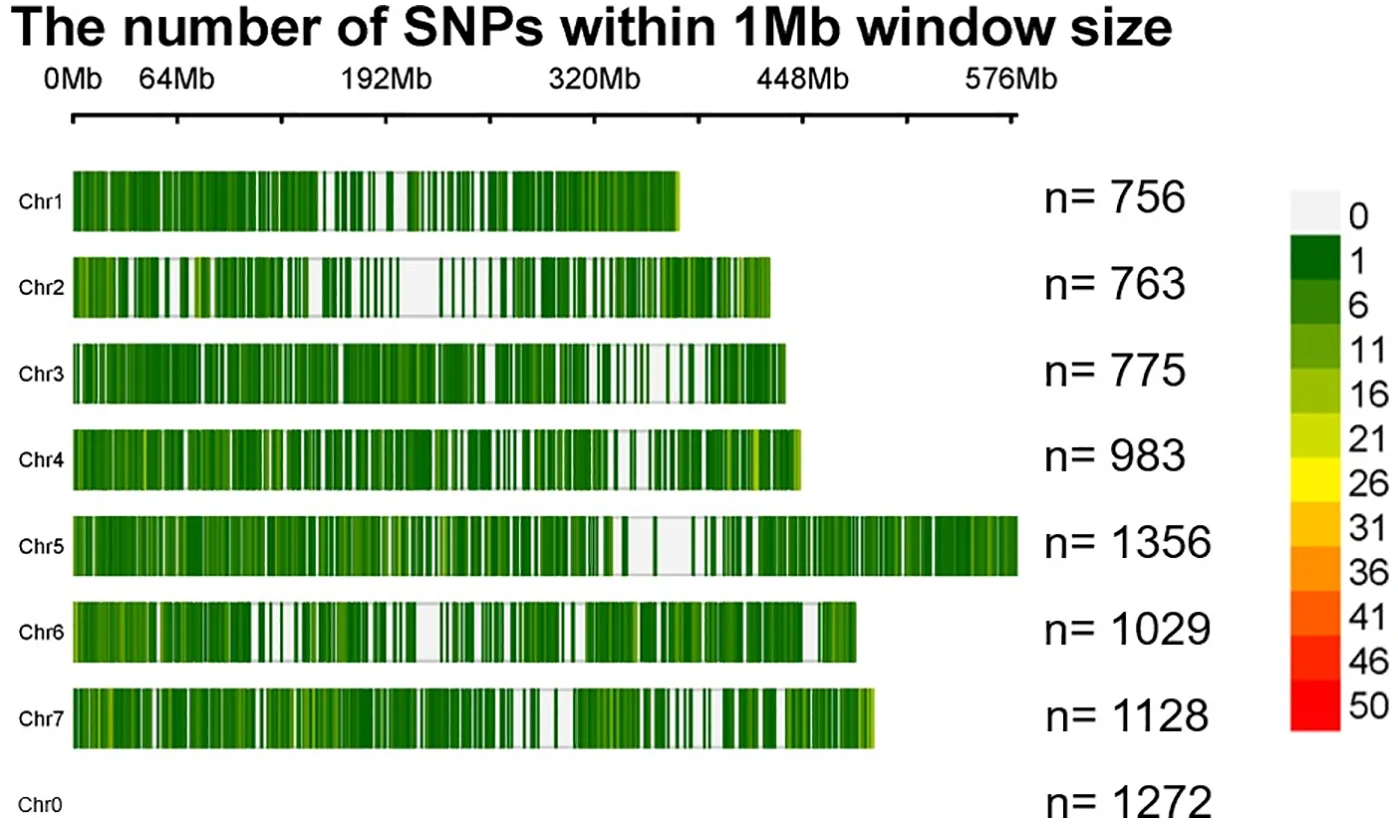
Distribution of SNPs on the pea genome. Following quality control, the SNPs that were chosen for the population structure analysis were mapped onto the pea genome. The Mb (megabases) bar at the top of the figure illustrates the position on the chromosomes, zero-indexed from 0 to 576 Mb. The reference genomes used by DArT are Pisum_sativum_ZW6_1.0 and Pisum_sativum_v1a. ‘Chr1-7’ represents the seven chromosomes of *Pisum sativum*, while ‘Chr0’ represents the SNPs that were not mapped to a location on the genome. ‘n=’ illustrates the number of SNPs mapped to each chromosome, e.g., 1,356 SNPs were mapped to Chromosome 5. The color of the heatmap illustrates the level of overlap of SNPs. As seen by the green color, the level of overlap was very low.

### Within-accession variation, and the mutant populations

The homogeneity of seed accessions was evaluated by calculating the within-accession genetic diversity by genotyping ten individuals from each of the ‘NGB 101304’ and ‘NGB 101997’ accessions obtained from NordGen. In the case of ‘NGB 101304’, the ten individuals showed genotypes sharing 93–98% of SNPs, with an average of 96% and a standard deviation of 0.015. The 10 seeds of ‘NGB 101997’ shared 91% − 98% of SNPs, with an average of 96% shared SNPs and a standard deviation of 0.02. Additionally, two mutant populations were discovered within the study population. 26 NordGen accessions shared on average 93.7% (SD = 1.12) of SNPs with each other. Most of these had “Weitor” listed in their pedigree. Six accessions with “Parvus” pedigree shared on average 94.2% (SD = 0.98) of SNPs. Many of the accessions in the mutant populations had a mutagen keyword included in their GeNBIS data records, confirming them as being parts of mutant populations. Including the mutant populations, and the 2x 10 accession individuals would lead to sampling bias and overestimation of the impact of the accessions in question. Therefore, both the accession individuals and the mutant populations were removed from the main analysis, so that only one individual per accession was included.

### STRUCTURE analysis, PCoA plot and population genetic indices

STRUCTURE analysis was performed to determine the number of subpopulations. The ‘Best K-by-Evanno’-plot revealed a major peak at K = 2 (Fig 2). A smaller peak appeared at K = 3. To explore this population structure, a PCoA plot was constructed visualizing the subpopulation structures at K = 3 (S3 File). Additionally, the individual accession assignments were recorded (S1File, column L). Changing the subpopulation number to three assigned all modern cultivars into the newly formed subpopulation 3. Some, but not all, landraces also distributed into the new subpopulation 3. Based on these results (S3 File), and the higher peak at K = 2, analysis of three subpopulations was not deemed more informative than two subpopulations. No further work with K = 3 was conducted. The analysis was continued with the assumption of K = 2. Based on this assumption, a PCoA plot was constructed to visualize the STRUCTURE analysis results. An accession was assigned to a subpopulation if it shared more than 80% of its SNPs with that subpopulation. Increasing and lowering the subpopulation membership definition (70 and 90% shared SNPs) did not change the subpopulation assignment for any accessions of subpopulation 2. Changing the cutoff altered subpopulation 1 assignments: the higher cutoff (90%) shifted more subpopulation 1 accessions to “admixed,” while the lower cutoff (70%) reassigned more admixed accessions to subpopulation 1. Additionally, a single accession (NGB 102999) changed from the ‘admixed’ assignment to subpopulation 2. PCoA plots following the 70% and 90% assignment definition were presented side-by-side with the 80% assignment, which was used in the final analysis (S4 File). The 80% assignment cutoff was chosen as it most clearly matched the subpopulation position with the location in the PCoA plot. It could be speculated if NGB 102999 share some ancestry with accessions related to subpopulation 2. The final subpopulations and the admixed individuals were visualized in the PCoA plot (Fig 3). Subpopulation 1 (Fig 3; *blue*) comprised all modern cultivars, along with many breeding and research lines, European landraces, and garden peas, totaling 184 accessions. Subpopulation 2 (Fig 3; *orange*) included all accessions of *Pisum sativum* subsp. *abyssinicum* and landraces from India and Nepal and consisted of 16 accessions. Finally, a group of accessions (Fig 3*; yellow*) were admixed, with <80% SNP similarity to either of the two subpopulations. This group was represented by 79 accessions. Some landraces were placed in the admixed group. The distance between subpopulation 2 and the remainder of the population was considerable (Fig 3). The clustering of the subpopulations was verified by a PCA visualization at both two and three subpopulations (S5 File). The overall division between subpopulations 1 and 2 was the same. The PCA revealed considerable division between two groups in subpopulation 2. Upon further inspection, the *Pisum sativum* subsp. *abyssinicum* accessions formed one group, and Indian and Nepalese landraces the other. This was only visible on the axis describing the second principal component (13.96% of variation vs. 35.3% for the first principal component). Despite being clearly divided on one axis, those two groups remained in the same subpopulation at K = 3. Therefore, it was supported to continue analysis with the assumption of two subpopulations.

**Fig 2:**
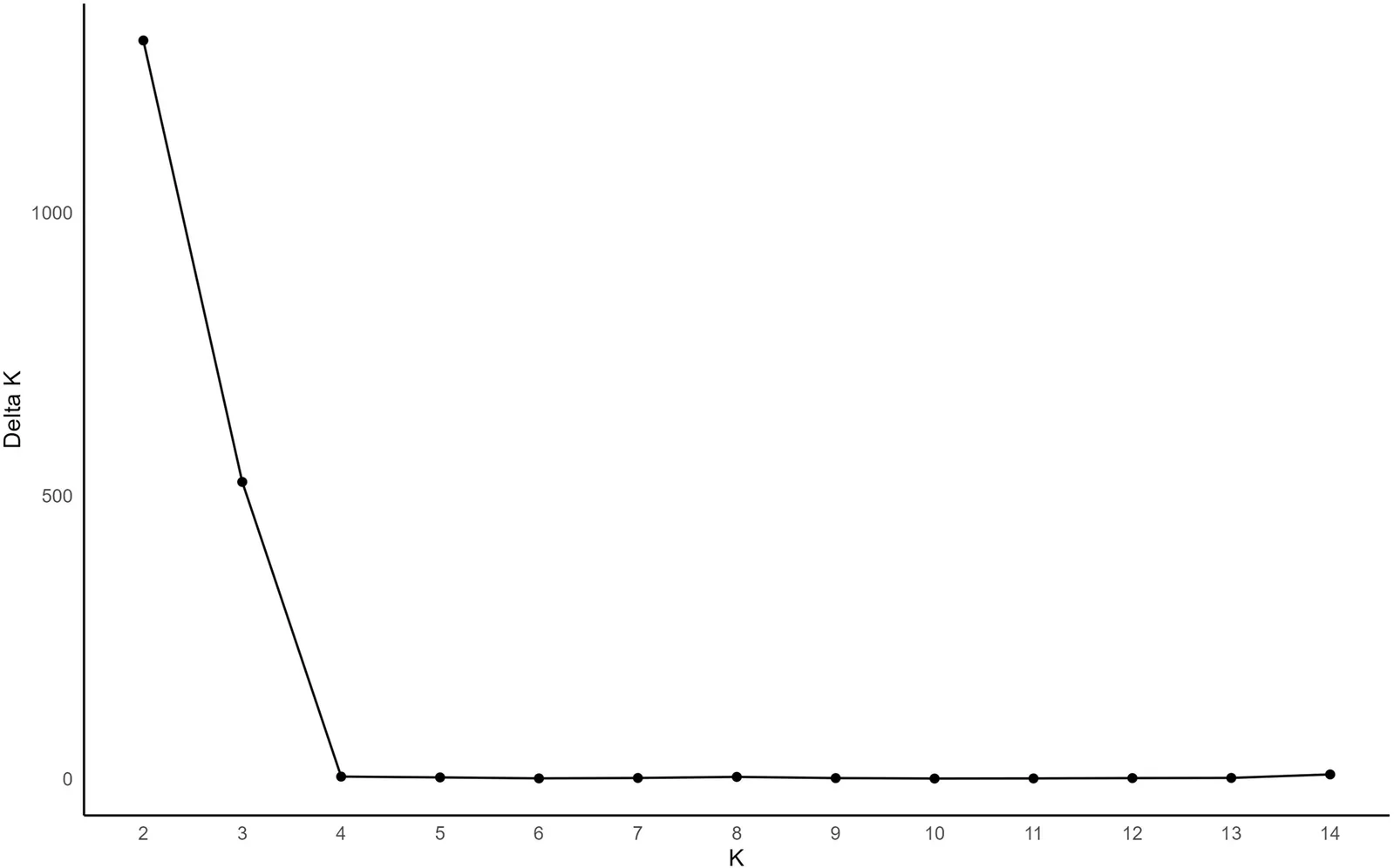
The ‘Best ΔK by the Evanno method’. Plotted using the DArT SNPs and the population of 279 Pea accessions. The number of potential subpopulations is indicated by ‘K’ on the x-axis, and the delta-K values on the y-axis.

**Fig 3:**
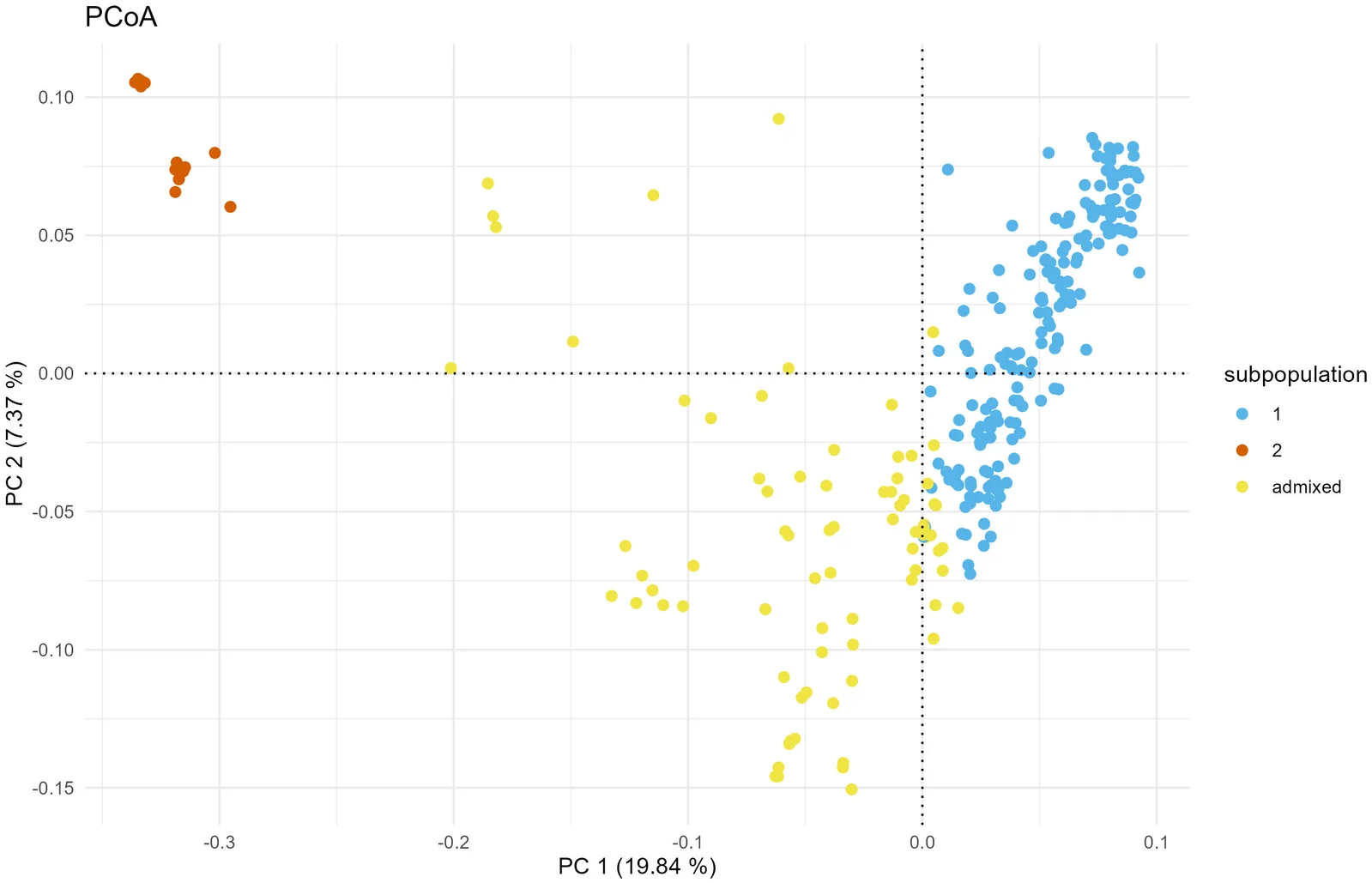
PCoA plot representation of the population of 279 pea accessions in the study, using DArT-seq based SNPs. Each dot represents an accession. The dot color indicates the subpopulation of that individual, as defined by the STRUCTURE analysis. Blue dots represent members of subpopulation 1, containing modern cultivars, breeding and research lines, and garden cultivars. Orange dots represent members of subpopulation 2 and include the subspecies *abyssinicum* and Indian and Nepalese landraces. The yellow dots represent the admixed individuals not belonging to any of the subpopulations. Only the first two principal components are shown.

To investigate the genetic dynamics of the subpopulations, various genetic diversity indices were calculated. The observed heterozygosity (Ho) and expected heterozygosity (He) of all subpopulations were estimated to calculate the inbreeding coefficients (Fis) of each subpopulation. As Fis values were greater than 0 for all subpopulations, the inbreeding nature of peas is confirmed. The differences between subpopulations in Ho, He and Fis were minute. Including both the global population, each subpopulation, and the admixed group. Ho ranged from 0.011–0.017, He from 0.161–0.330, and Fis from 0.932–0.948 (S6 File).

To estimate the genetic distance between the subpopulations, the pairwise fixation index (Fst) was calculated. Fst values closer to zero indicate complete interbreeding between subpopulations, while values closer to one indicate complete subpopulation separation [39]. The Fst between subpopulations 1 and 2 was 0.497, between 1 and the admixed group was 0.099, and between subpopulation 2 and the admixed group was 0.366 (S7 File).

### The neighbor-joining trees

The neighbor-joining analyses resulted in trees with high levels of branching and long branch lengths (Figs 4 and 5). The NJ-trees showed many monophyletic groups of closely related accessions (Figs 4 and 5). The orange subpopulation from the PCoA analysis (Fig 3) was divided into two clades (Fig 4, *orange;* Fig 5, *purple and orange*). The innermost of the two clades included four *Pisum sativum* subsp. *abyssinicum* accessions, and four accessions labeled as “Traditional Cultivar or Landrace” in the GeNBIS database (Fig 4, *orange*; Fig 5, *purple;* S1 File). The second clade was resolved with longer branches between individuals and included the Indian and Nepalese landraces (Fig 4*, orange*; Fig 5, *orange;* S1 File). The remaining accessions included in this study were resolved as a monophyletic clade. This clade included both subpopulation 1 from the STRUCTURE analysis (blue), and the admixed accessions (Fig 4*, yellow*). For the accessions in which multiple seeds were included, NGB 101304 (Fig 4*, A*), and NGB 101997 (Fig 4*, B*), the branch lengths between individuals were short. The modern cultivars were resolved as a monophyletic group, supported by a bootstrap value of 99% (Fig 4*, C;* Fig 5 *blue overlay*). These modern accessions represented agricultural cultivars released after 2000, as well as two gene bank accessions, ‘NGB 22640 Trille’ and ‘NGB 20117 Lollandske rosiner’ (Fig 5, *blue overlay*). Four morphological traits of agronomic importance varied in how they matched the clades in the NJ tree (Fig 5, *columns*). 1) Absence of leaflets and presence of tendrils, “*afila”,* was present in all modern cultivars and in 12 accessions of mostly Swedish breeding or research lines (Fig 5, *first column, green*). 2) When the agronomic trait, thousand-seed-weight, was mapped onto the NJ tree, the modern cultivar clade showed large seeds with an average thousand-seed-weight of 241 ± 40 g (mean + SD, n = 21), in contrast the *Pisum sativum* subsp. *abyssinicum* clade was characterized by small seeds averaging just 71 ± 30 g (mean + SD, n = 16). The overall population mean was 172 ± 57 g (mean + SD, n = 279). (Fig 5, *second column, large seeds; dark color, small seeds; pale color*). 3) The majority of the population, 170 accessions, had the white-flowered phenotype, while 48 accessions with purple flowers were resolved throughout the NJ tree (Fig 5, *third column, purple and white*). The *Pisum sativum* subsp. *abyssinicum-*clade, The Indian and Nepalese clade, and some *Pisum sativum* var. *elatius*, and some *P. sativum* labeled in GeNBIS as “wild” were mostly purple-flowered. All modern cultivars, except cultivar ‘Rainbow’ had white flower morphology. Six accessions had white-and-pink flowers while five accessions had red flowers (Fig 5, *third column, pink and red*). 4) When the morphological trait, “pea surface” was mapped onto the NJ tree, about half of the accessions were of the smooth-surface type, and the other half were wrinkled (Fig 5, *fourth column*). Most modern cultivars and all accessions in the *Pisum sativum* subsp. *abyssinicum-*clade were of the smooth phenotype. To validate associations between phenotypes and subpopulations, χ^2^ independence analysis and Pearson residuals were analyzed (Table 2). Flower color and pea surface type showed association to subpopulations (χ^2^ p-value <0.001 for both). The white flower morphology was over-represented in subpopulation 1 (Pearson residual = 2.6), and the pigmented flower colors were under-represented (Pearson residual = −4.5). The opposite was the case in subpopulation 2 where pigmented flowers were over-represented, and white under-represented (Pearson residuals = 4.9 and −2.7). Both support visualizations in Fig 5. The wrinkled surface type was over-represented in subpopulation 1 (Pearson residual = 2.8) while the smooth was over-represented in subpopulation 2 (Pearson residual = 2.5). Leaf morphology did not show significant association to subpopulations (χ^2^ p-value = 0.18) (Table 2).

**Table 2:** Result summary of the χ^2^ test of independence and related Pearson residuals. P-values and Pearson residuals presented at each phenotype of each trait for both subpopulations and the admixed group. Significant p-values (p < 0.001) indicates that the observed phenotype frequencies had significant association to subpopulation structure. Pearson residuals indicate if the specific phenotype correlated with specific subpopulations. Positive values indicate overrepresentation of a phenotype within a subpopulation, while negative values indicate underrepresentation. Pearson residuals should not be interpreted if the χ^2^ p-value is above the significance level.

Trait	Subpopulation	Phenotype	Pearson Residual	Chi^2^ p-val
Flower color	1	pigmented	−4.51	1.99 x 10^–20^
		white	2.56	
	2	pigmented	4.87	
		white	−2.77	
	admixed	pigmented	4.95	
		white	−2.82	
Surface type	1	smooth	−2.60	1.11 x 10^−10^
		wrinkled	2.85	
	2	smooth	2.46	
		wrinkled	−2.70	
	admixed	smooth	2.83	
		wrinkled	−3.10	
Leaf morphology	1	afila	0.76	0.18
		wild-type	−0.31	
	2	afila	−1.45	
		wild-type	0.59	
	admixed	afila	−0.53	
		wild-type	0.21	

**Fig 4:**
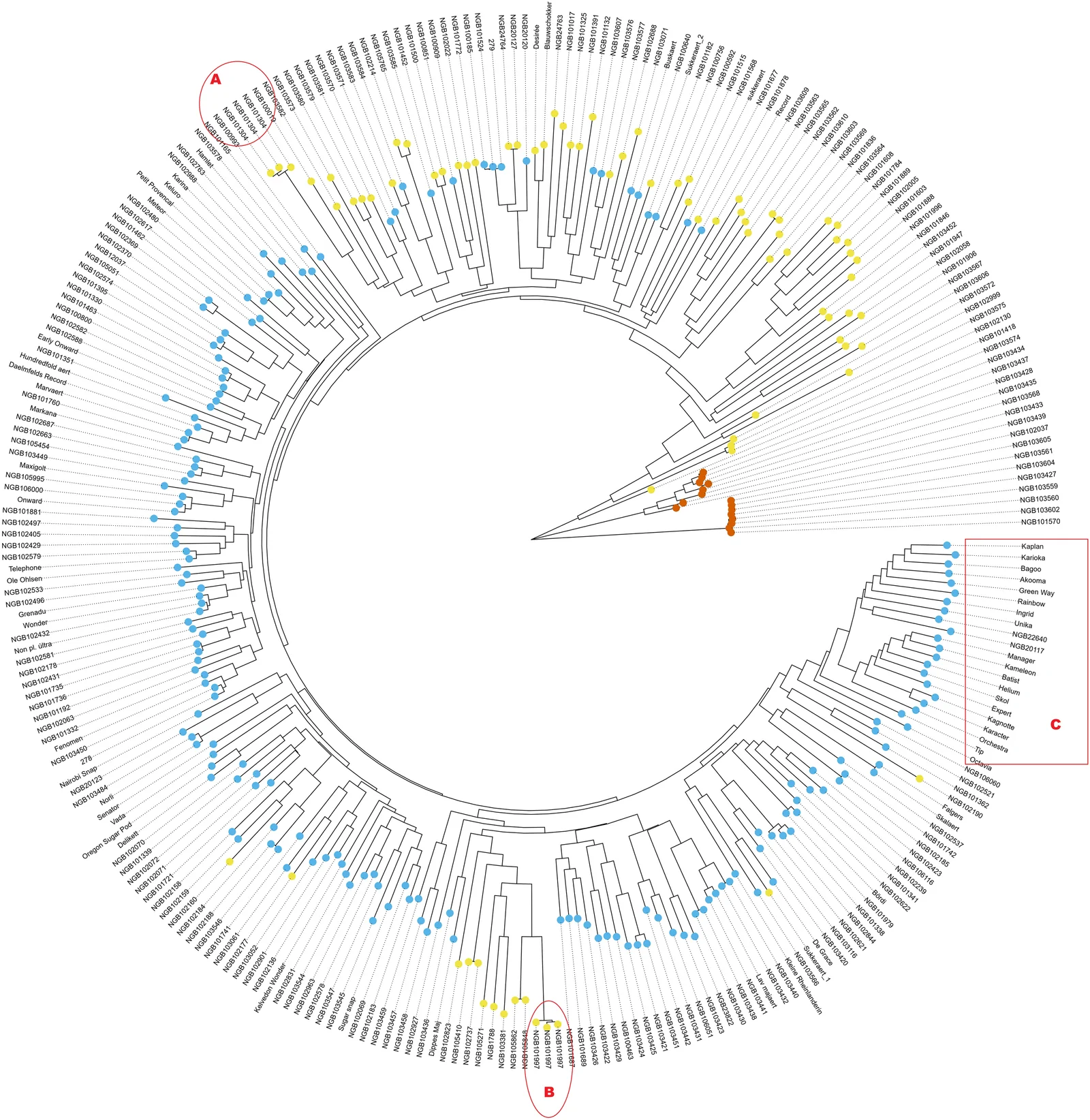
A circular Neighbor Joining tree of the 279 accessions of Peas in the study. The names on the outer circle are either the NordGen accession number (from The Nordic Genebank), or the cultivar/landrace/other name whenever available (S1 File). The colored dots represent subpopulations defined by the STRUCTURE analysis. Blue dots represent members of subpopulation 1 which includes modern cultivars, breeding and research lines, and garden cultivars. Orange dots (subpopulation 2) represent accessions belonging to the *Pisum sativum* subsp. *abyssinicum* or Indian and Nepalese landraces-group. The yellow dots represent the admixed individuals that do not belong to any of the subpopulations. The letter A highlights individuals of accession NGB 101304, B highlights individuals of accession NGB 101997, and C highlights the modern cultivars.

**Fig 5:**
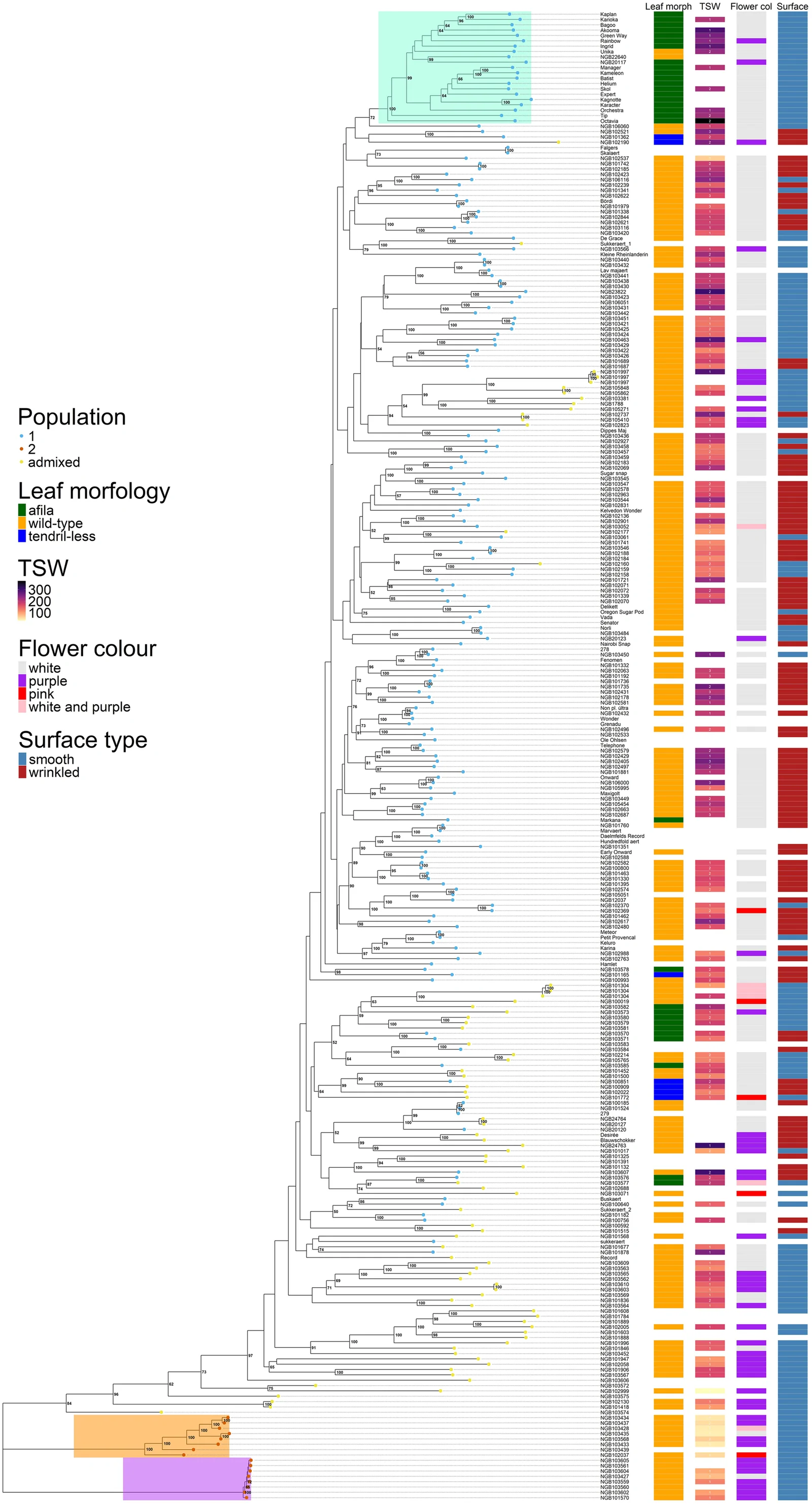
A linear Neighbor-Joining tree of the 279 accessions of peas in the study, including selected morphological traits. The tree was based on the DArT-seq-based SNPs. The overlaying colors on the tree were manually placed to draw attention to three groups. The purple overlay covers the *Pisum sativum* subsp. *abyssinicum* accessions. The orange covers the Indian and Nepalese landraces. The blue overlay covers the modern cultivars. At the terminal end of each branch is a colored dot representing the subpopulation defined by the STRUCTURE analysis. The blue dots symbolize members of subpopulation 1, which contain modern cultivars, breeding and research lines, and garden cultivars (Figs 3 and 4). Orange dots (subpopulation 2) are accessions belonging to the *Pisum sativum* subsp. *abyssinicum* or Indian and Nepalese landrace group. The yellow dots are the admixed individuals not belonging to any of the subpopulations. Each accession ID or cultivar name is presented at the end of the dotted line extending from each branch. The numbers at branching points depict bootstrap values >50. The four columns represent the accessions’ morphologies in four traits. Missing data is represented by no fill. Column one displays the leaf morphology (tendrilless (blue), wild-type (yellow), and *afila* (green). The second column displays the thousand-seed-weight using a color gradient ranging from yellow (100 g) to dark purple (300 g). The thousand-seed-weight are averages from several years of field experiments described in Winther et al. (2023). The white number in the columns indicate the number of years of data used to calculate the color of the gradient for each accession. The third column shows the flower color: white flower (grey), purple flower (purple), pink flower (red), or white-and-pink flower (light pink). The last column depicts the surface type of the accessions: smooth surface (blue), and wrinkled surface (red).

## Discussion

### DArT genotyping of *Pisum sativum*

A limited number of studies have applied the DArT genotyping platform to pea populations. After careful consideration, we adopted the stringent data filtering criteria imposed by others [20]. Our quality control left 8062 DArTseq SNPs. This is approximately 30% fewer than reported in [20], resulting in a similar reduction of chromosome coverage. However, as visible in Fig 1, no large areas of the genome were uncovered by SNPs. Additionally, our NJ-tree and PCoA analyses resulted in high levels of branching and accession separation. These results supported the robustness of our data filtering approach and led us to support and replicate high standards of data curation for this recently developed genetic diversity method.

### Within-accession genetic similarity

We genotyped 10 individuals from two accessions to assess genetic similarity of seeds from single accessions. The mean genetic similarity was 96%, ranging from 91% to 98%.

A similar study investigates the variation in individuals of modern cultivars of soybean. It concludes that a 96% identity threshold is a reasonable, albeit conservative measure for SNP-based distinction between individuals of an approved soybean cultivar in the ‘Distinctness, Uniformity, and Stability’ (DUS) system for cultivar recognition [40]. Based on our results we recommend that a lower threshold should be established for gene bank accessions, as many of our individuals fell below the 96% threshold suggested for soybean cultivars. Based on the two accessions, an identity threshold could be suggested based on the upper 95^th^ quintile of individual similarities. For NGB101304 the upper 95^th^ quintile was at 94%, and for NGB101997 it was 93%. Those thresholds might be used until more thorough results can be achieved within this field. It is likely that higher levels of diversity exist within other accessions, which would mean that population studies like ours misrepresent individual accessions. Additionally, this has consequences for the conservation of accession diversity under gene bank management. Within-accession diversity likely varies between species, due to differences in pollination biology, inbreeding degrees, and other species-specific characteristics. This means that accession identity thresholds should be established for each species individually. Conclusively, more investigation should be done on the genetic diversity within individual gene bank accessions.

### Our results in the context of “The K = 2 conondrum”

A disproportionate number of studies may be falsely arriving at K = 2 conclusions for subpopulation estimates. This tendency has been named the “K=2 conondrum” [41]. Based on the ‘Best-K by the Evanno-method’, we performed our analysis on the K = 2 hypothesis. With the “K=2 conondrum” in mind, it was relevant to discuss the reliability of our method. One way to validate the K hypothesis is to consider more methods than only the Evanno method [27,41]. In our case, we used the neighbor-joining tree, where we saw a clear division between subpopulation 2 and the rest of the accessions (Figs 4 and 5). Additionally, we supported our K = 2 hypothesis by computing PCA and PCoA at K = 2 and K = 3 (S3, S5 Files). All admixed and subpopulation 1 accessions shared a common root, while subpopulation 2 seemed to have two roots. As such, based on the tree, subpopulation 2 could be divided into two subpopulations: one including the *Pisum sativum* subsp. *abyssinicum* accessions, and one containing the Indian/Nepalese accessions (Fig 5; *purple, orange and blue overlay*). Based on our PCA and PCoA analyses at K = 3 we conclusively continued with K = 2.

### The subpopulations

Both landraces and modern cultivars were resolved in subpopulation 1 and the landraces were distributed throughout the subpopulation and the admixed subpopulation. This underlined the narrow diversity amongst the modern cultivars. In contrast, the landraces were not closely related as they were not organized in groups of any kind. The fact that most of the population was organized in subpopulation 1 indicated high genetic relatedness of the population. This likely stems from a shared northern European origin. Subpopulation 2 contained the *Pisum sativum* subsp. *abyssinicum* accessions, as well as Indian and Nepalese landraces. Considering the geographical distance between these accessions and the rest of the population, this was very reasonable. To clarify the differentiation between subpopulations, we calculated Fst values. The Fst values of 0.497 and 0.366 signified high population differentiation between subpopulation 2 and both subpopulation 1 and the admixed group. The geographic origin of the accessions of subpopulation 2 was Northern Africa, India and Nepal, and the high population differentiation implied a reduced import of genetic material from these regions into the regions of subpopulation 1 (mostly Scandinavia and northern Europe). In contrast, the low Fst value of 0.099 indicated that the admixed group was most closely related to subpopulation 1. Several highly admixed subpopulations were also found and described by Rispail et al. (2023) who suggested taxonomic subdivisions of pea subspecies based on their results. Our study did not include a wide range of subspecies, and as such cannot support or refute those conclusions. Rather, our results underline that even the admixed population is more closely related to European genetic sources than to Northern African, Indian or Nepalese.

In population structure studies of peas, geography often correlates well with subpopulation structures. For example, another study finds that *Pisum sativum* subsp. *abyssinicum* and other wild subspecies cluster together, distinctly from *P. sativum subsp. Sativum* [20]. Furthermore, they identify another distinct cluster containing Indian landraces [20]. The separation of Indian/Nepalese accessions and *Pisum sativum* subsp. *abyssinicum* accessions found by Rispail et al., (2023) might be due to the larger number of subspecies included, since the genotyping platforms used are identical, and cannot explain the difference. Another study finds six subpopulations. One of those contain landraces from Afghanistan, China, India, Iran, Nepal, and Pakistan. Another contains accessions of mainly Indian and Ethiopian origin, which again aligns with our results [42]. When our analysis was unable to find additional subpopulations, it might indicate the relatively homogeneous history of the studied accessions. A large part of our population originated from the Weibullsholm collection [43,44]. Weibullsholm accessions are labeled with a WBH-number (S1 File) and in NordGen’s database. This indicated a shared history between many of the breeding/research lines of this origin, which would explain the lack of subpopulations. Additionally, most of the lines from the Weibullsholm-collection included in this study are listed as having Scandinavian, primarily Swedish origin. As such, our population did not represent the seven agro-climatic zones of Europe, from above the polar circle to the Mediterranean Sea evenly. If our population had included, for example, landraces of southern or central European origin, it is possible that we would have found subpopulation structures matching that geography. There was very likely to be a bias due to the geographic origin of many of the accessions. Because our focus was on the northern European population structures, care should be taken before generalizing our findings.

### Diversity in modern cultivars vs. landraces and the importance of gene banks

The NJ-tree showed that all modern cultivars in the population were located in a single clade (Fig 5, *blue overlay*). This implied narrow genetic diversity between the modern cultivars relative to the much larger genetic diversity across the population. In contrast to the genetic homogeneity of the modern cultivars, the landraces and garden peas did not cluster together. Instead, they were distributed throughout the tree. This may partly be due to the different cultural, culinary, and agronomic uses of the landraces; e.g., the diversity of types and cultivars within the garden peas which include snap, shelling, and marrowfat types. Tran et al. (2022) investigate the genetic diversity of 46 pea cultivars and finds that two clusters are formed mainly based on the presence or absence of the *afila* phenotype. Tran et al. (2022) conclude that the genetic diversity in the subpopulation displaying the *afila* phenotype is smaller than that of the wild-type phenotype. Furthermore, they speculate that historical accessions with wild-type leaf morphology could be an important source of genetic resources for future breeding [45]. Although our terminology was different, our results were in accordance with those of Tran et al. (2022). It can be assumed that the *afila*-cluster described by Tran et al. (2022) represents what we called “modern cultivars”. The low diversity among the modern cultivars in our study mirrors the low diversity of the *afila-*cluster [45]. The clustering of the modern cultivars in that [45] and our study confirm similar conclusions reached with now-outdated methods: that modern cultivars display less genetic diversity in general [46], and that western European breeding programs specifically are based on a narrow genetic basis [47]. Although this may be regarded as a well-known fact, such common knowledge needs to be tested experimentally as in this study, to show the magnitude of erosion of genetic diversity in the specific crop, pea. The pea population in this study thus contribute to elucidate the potential of the embedded genetic diversity within the species for future breeding programs. During the 1960’s, the Green Revolution fostered widespread distribution and adoption of modern and high-yielding cultivars. The advancement of modern cultivars led to a drastic decrease in the cultivation of locally grown landraces. While modern cultivars were one of the key reasons for the yield-increases documented from the 1960’s and onwards, the wide-scale cultivation of crops with a narrow genetic basis led to an overall decrease in system resilience [48]. The detrimental results of large-scale cultivation of individual cultivars have been demonstrated multiple times during history, for example with the southern corn leaf blight epidemic of 1970s [49], and the Irish famine caused by the collapse of potato production caused by *Phytophthora infestans* in the 1840’s [50]. These and similar epidemics facilitated the realization that plant genetic resources can be a crucial source of disease resistance in crops [51]. The low level of diversity in modern cultivars relative to the total diversity emphasizes the importance of gene banks as widely recognized in the literature [52,53]. Care should be taken to ensure that the diversity preserved in landraces and historical accessions is not lost through ‘genetic erosion’. This is a possible risk when local landraces are neglected for modern high-yielding cultivars [48]. Effective conservation starts with knowledge. Recognizing genetic uniqueness, as well as genetic duplicates, is important for the proper management and utilization of genetic resources. Studies like ours contribute to this by mapping, characterizing, and genotyping the available germplasm, thereby supporting the global identification and documentation of plant genetic resources. This is achieved by making the genotype dataset public (https://doi.org/10.5061/dryad.dr7sqvbdw).

### Ideotypes and morphological traits

To gain insights into the developmental history of the 279 pea accessions, the linear NJ-tree was aligned with important morphological traits relevant to pea breeding and ideotype definition. The *afila*-mutation was first described in 1953 and has since been introgressed into virtually all modern cultivars. The *afila* phenotype leads to improved harvestability, lower susceptibility to foliar diseases, and ultimately higher yields. The *afila* phenoype has therefore been incorporated into breeding programs worldwide [22]. The *afila*-phenotype was present in almost all modern cultivars in our population. The exceptions from this rule were cultivars NGB 22640 (‘Trille’) and ‘Unika’, which displayed the wild-type leaf morphology, despite being members of the cluster of modern cultivars. Both accessions, however, are historical cultivars from before 2000. NGB 22640 (‘Trille’) is a cultivar registered in Denmark in 1987 [54], and ‘Unika’ is a cultivar grown as early as 1929 [55]. While the *afila*-phenotype was present in almost all the modern cultivars, it was not present in all individuals of subpopulation 1. This was underlined by the lack of significant association between leaf morphology and subpopulations as indicated by a χ^2^ p-value >0.05. This may be due to the monogenetic background of the phenotype. Considering thousand-seed-weight, the modern cultivars displayed mostly large seeds, with an average of 248 g for the modern cultivars compared to the population mean of 175 g. In contrast, the *Pisum sativum* subsp. *abyssinicum* as well as the Nepalese and Indian group displayed mostly small seeds with an average seed weight of just 77 g. Similarly to the dominance of the *afila*-phenotype in modern cultivars, this also reflects the demand for large seeds in modern ideotypes [46,56]. Furthermore, all the modern cultivars investigated in this study had white flowers, except for cultivar ‘Rainbow’. In contrast, the majority of the garden peas and landraces displayed purple-flowered morphology. Additionally, among the basal branches of the NJ-tree, most of the accessions in the *Pisum sativum* subsp. *abyssinicum*/Nepalese/Indian population had purple flowers. These observations were supported by the χ^2^ analysis and the Pearson residual values indicating the association between subpopulations and flower colors. In our study, all accessions in the *Pisum sativum* subsp. *abyssinicum* and Nepalese/Indian landrace groups were of the smooth seed type. All the modern field pea cultivars also had smooth seeds. Again, these correlations were confirmed with the associations indicated by the χ^2^ analysis. An ideotype is described for a French environment, where the overall goal is yield stability through traits leading to high yields, reduced lodging, and resistance to biotic and abiotic stresses. Notably, large seeds, high protein content, flowering date and length, plant height, and lodging resistance are described [56]. Except for plant height, those traits were especially present in the modern cultivars in our study. Similarly, molecular markers have been used to trace important breeding goals such as high seed weight, the *afila* leaf phenotype, and white flowers, as well as cold tolerance in winter peas [46]. The breeding goals for Scandinavian pea breeders are, among others, yield, lodging resistance, earliness, quality, disease resistance, protein concentration, seed color, shape and size [57]. Modern plant breeding depends on genotypic selection and would benefit from genotyped germplasm collections such as those described in this study. Thus, publication of genomic and phenotypic data can aid pea breeders globally in general, and in Scandinavia specifically, and making such data publicly available should be encouraged.

This study emphasized the value of germplasm management, which focuses on maintaining genetic diversity for cool climates such as Scandinavia. Additionally, the data presented has value in a pre-breeding context. The genomic, diversity and phenotypic data in this study provides valuable datasets for pea breeding enabled by artificial intelligence (AI). The information may be taken into account in the outline of future pea improvement programs for quality and agronomic traits. This has recently been accomplished for abiotic stress tolerant peas [58].

## Conclusions and perspectives

This study is unique in its inclusion of modern cultivars. These represent several breeding companies, as well as some very recent-to-market cultivars, which, to our knowledge, are not investigated in other studies. It has become clear that the modern cultivars selected for our study are closely related relative to the rest of the population, as they are organized in a monophyletic cluster on the NJ-tree. In contrast, the landraces and the garden peas are dispersed throughout the NJ-tree with no real structure. Overall, the population consists of two subpopulations. At the basal branches of the tree, a subpopulation contains Indian and Nepalese landraces as well as accessions of *Pisum sativum* subsp. *abyssinicum*. All other accessions are resolved either in another subpopulation, or admixed. The level of diversity within the modern cultivars group is significantly smaller than that of the rest of the population.

Our study adds to the growing body of research investigating genetic diversity in various pea populations. The increasing number of genotyped accessions will allow for comprehensive structure analyses like the one conducted by Pavan et al. (2022), who combines SNP data from two germplasm collections, which allows them to analyze a total number of 652 accessions [42]. It is easy to imagine that, within a short time period, someone will compile the increasing number of SNP datasets, which will allow for one large-scale, global-coverage population, including all subspecies, landraces from many regions, and a large number of the modern cultivars in use globally. This will lead to a high level of understanding of global population structures, how genetics have been moved around the globe, and ultimately conclude the evolutionary relationships between the various domesticated *Pisum* subspecies. Our study reinforces the necessity of gene banks safeguarding germplasm, as modern northern European agriculture appears not to be a reasonable way for in situ conservation of agricultural genetic resources. We have found great genetic diversity in both traditional landraces, and garden peas, and so maintaining these types of accessions seems a reasonable way to maintain genetic diversity in *P. sativum*.

## Supporting information

S1 File An overview of all the accessions investigated in this study. Accessions are listed in the order that they appear on the Neighbor Joining tree (Fig 5). Part of the information is queried from NordGens database GeNBIS available at https://www.nordic-baltic-genebanks.org/gringlobal/search. Column A is the cultivar name, landrace name, or accession name as defined in the database whenever one is available. Column B is the NGB number. Column C is the WBH (Weibulsholm) number for genotypes originating from the Weibulsholm collection. Column D is the category as defined in GeNBIS, with the exception of the categories “modern cultivar”, “landrace”, “older cultivar”, “garden pea”, which is defined by the authors. “Modern cultivar” is used to describe cultivars developed after year 1990. “landrace” is used to describe accessions recorded before year 1945. “older cultivar” is used to describe cultivars older than year 1990, but younger than 1946. “garden pea” is used to describe pea accessions that are available for garden growers, with no sizeable commercial production. Column E is the country where the genotype was breed, collected or otherwise originate. These are likely to be inaccurate, as it is hard to pinpoint in many cases. Column F is the breeding company if available. Column G is the year of release for cultivars, or year of donation to NordGen if the year of release is not possible to pinpoint. Columns H, I, J and K are leaf morphology, thousand seed weight in 2023, flower colour and surface type as determined in our study. Column L is the subpopulation designation when following the K = 3 hypothesis.
(XLSX)

S2 File A picture of the single row field evaluation setup. Rows (and the picture) are facing west.
(ZIP)

S3 File PCoA visualization at K = 2 (Top) and K = 3 (Bottom). (PDF)

S4 File PCoA visualization with three levels of subpopulation assignment cutoffs. 70% identity cutoff (top left), 80% identity cutoff (right), 90% identity cutoff (bottom left). The 80% cutoff was the one used in the analysis.
(PDF)

S5 File PCA visualizations at K = 2 (top) and K = 3 (bottom). Subpopulation 1 remains closely organized, while the PCA splits subpopulation 2 into the *Pisum sativum* subsp. *abyssinicum* and Indian and Nepalese accessions (top and bottom right respectively).
(PDF)

S6 File Genetic statistical indices. Ho, He and Fis within each subpopulation and the admixed group.
(DOCX)

S7 File Pairwise Fst between each subpopulation and the admixed group. (DOCX)
