# Supplementary material for: Modern pea (Lathyrus oleraceus) cultivars exhibit significantly lower genetic diversity relative to landraces, garden cultivars and genebank accessions
Source: PLoS One. 2026 Oct 21;21(10):e0357113. doi: 10.1371/journal.pone.0357113 (PMC13686274; doi:10.1371/journal.pone.0357113)
Supplement: Supplementary file 1 [file pone.0357113.s003.pdf]

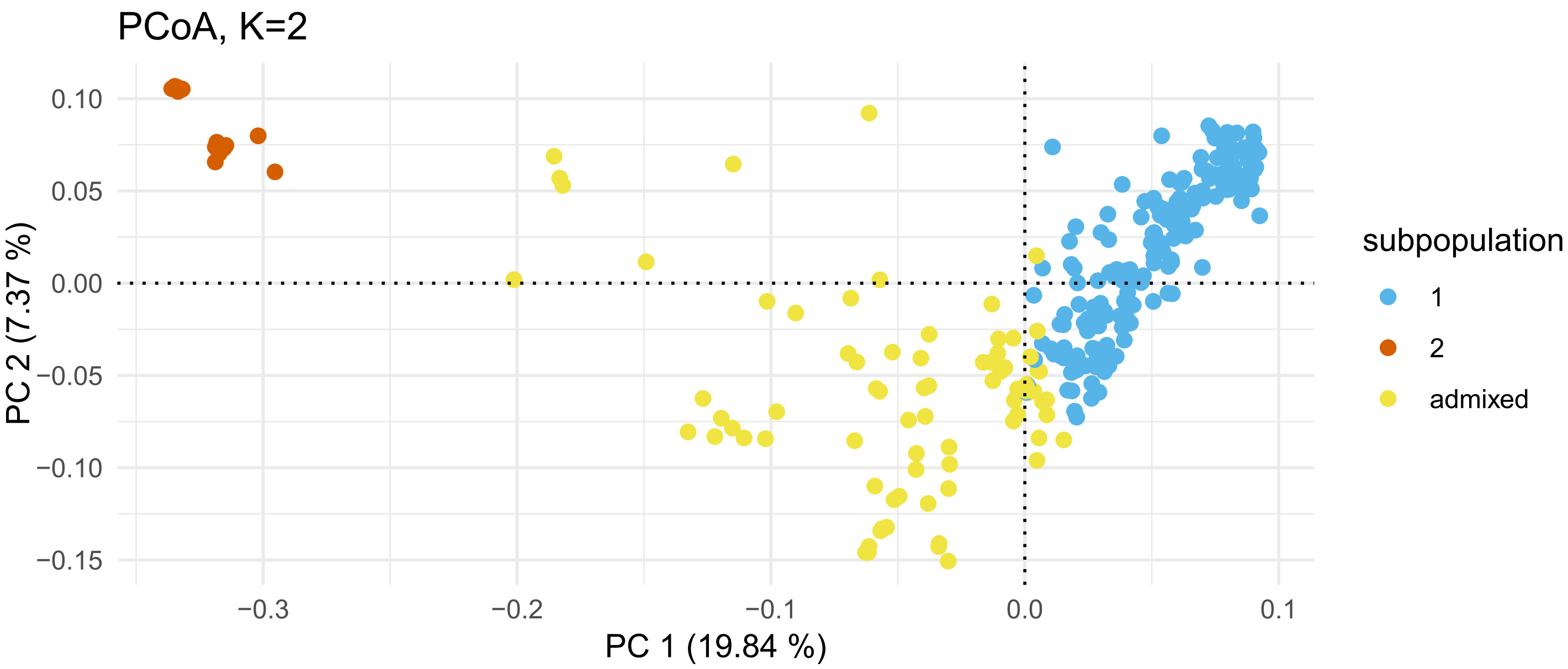
PCoA, K=2
0.10
0.05
0.00
−0.05
−0.10
−0.15
PC 2 (7.37 %)
−0.3
−0.2
−0.1
0.0
0.1
PC 1 (19.84 %)
subpopulation
1
2
admixed

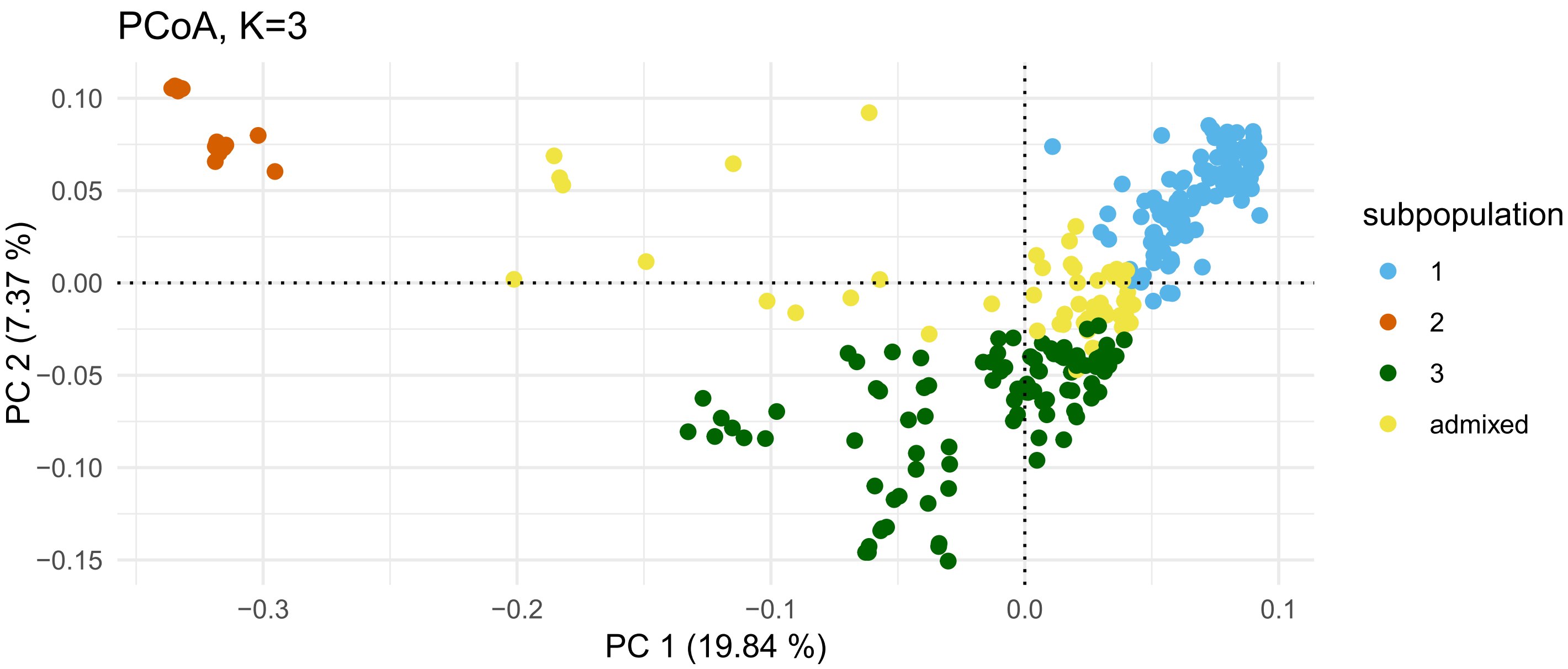
PCoA, K=3
0.10
0.05
0.00
−0.05
−0.10
−0.15
PC 2 (7.37 %)
−0.3
−0.2
−0.1
0.0
0.1
PC 1 (19.84 %)
subpopulation
1
2
3
admixed
