# Supplementary material for: Modern pea (Lathyrus oleraceus) cultivars exhibit significantly lower genetic diversity relative to landraces, garden cultivars and genebank accessions
Source: PLoS One. 2026 Oct 21;21(10):e0357113. doi: 10.1371/journal.pone.0357113 (PMC13686274; doi:10.1371/journal.pone.0357113)
Supplement: Supplementary file 2 [file pone.0357113.s004.pdf]

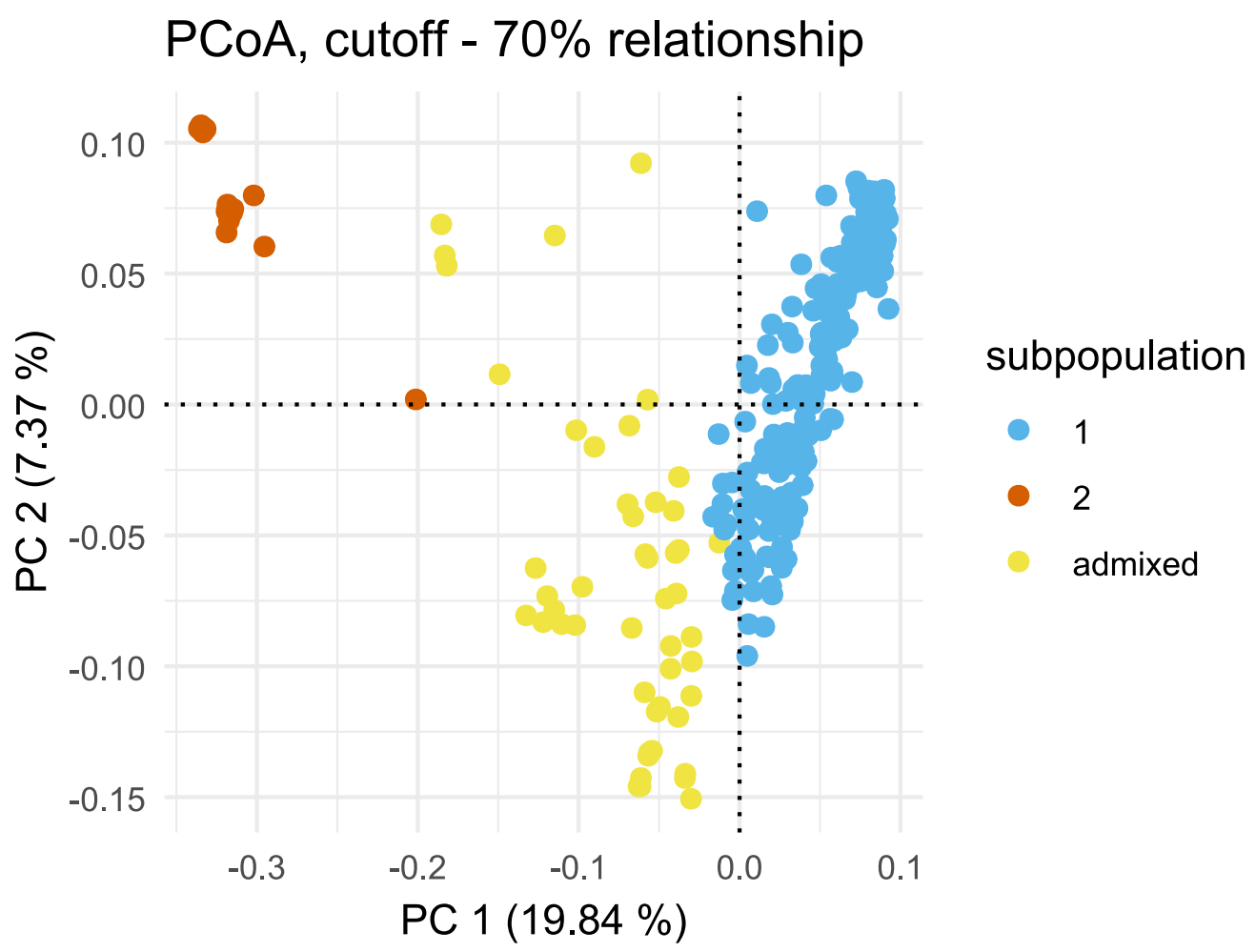

PCoA, cutoff - 70% relationship
0.10
0.05
0.00
-0.05
-0.10
-0.15
PC 2 (7.37 %)
-0.3
-0.2
-0.1
0.0
0.1
PC 1 (19.84 %)
subpopulation
1
2
admixed

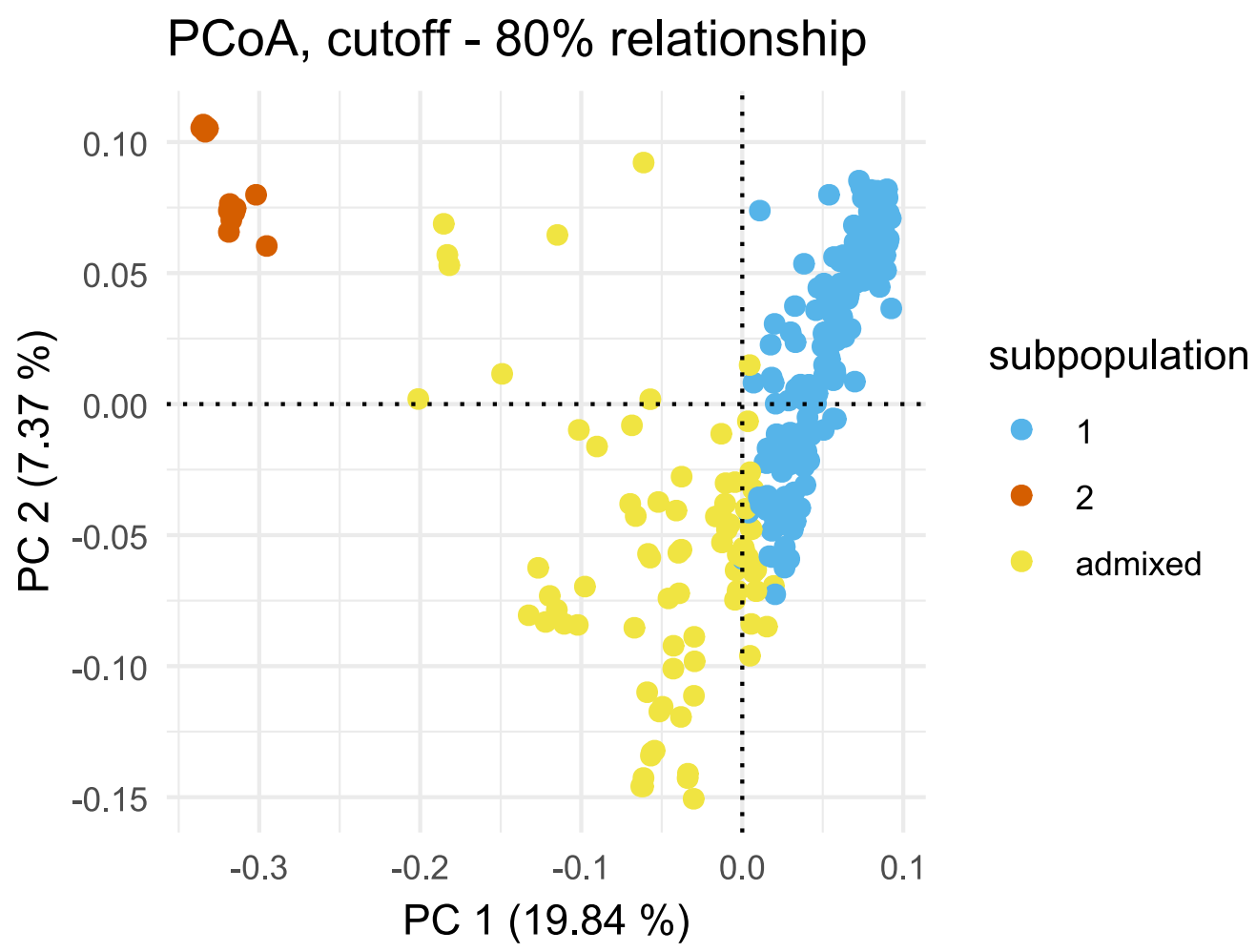

PCoA, cutoff - 80% relationship
0.10
0.05
0.00
-0.05
-0.10
-0.15
PC 2 (7.37 %)
-0.3
-0.2
-0.1
0.0
0.1
PC 1 (19.84 %)
subpopulation
1
2
admixed

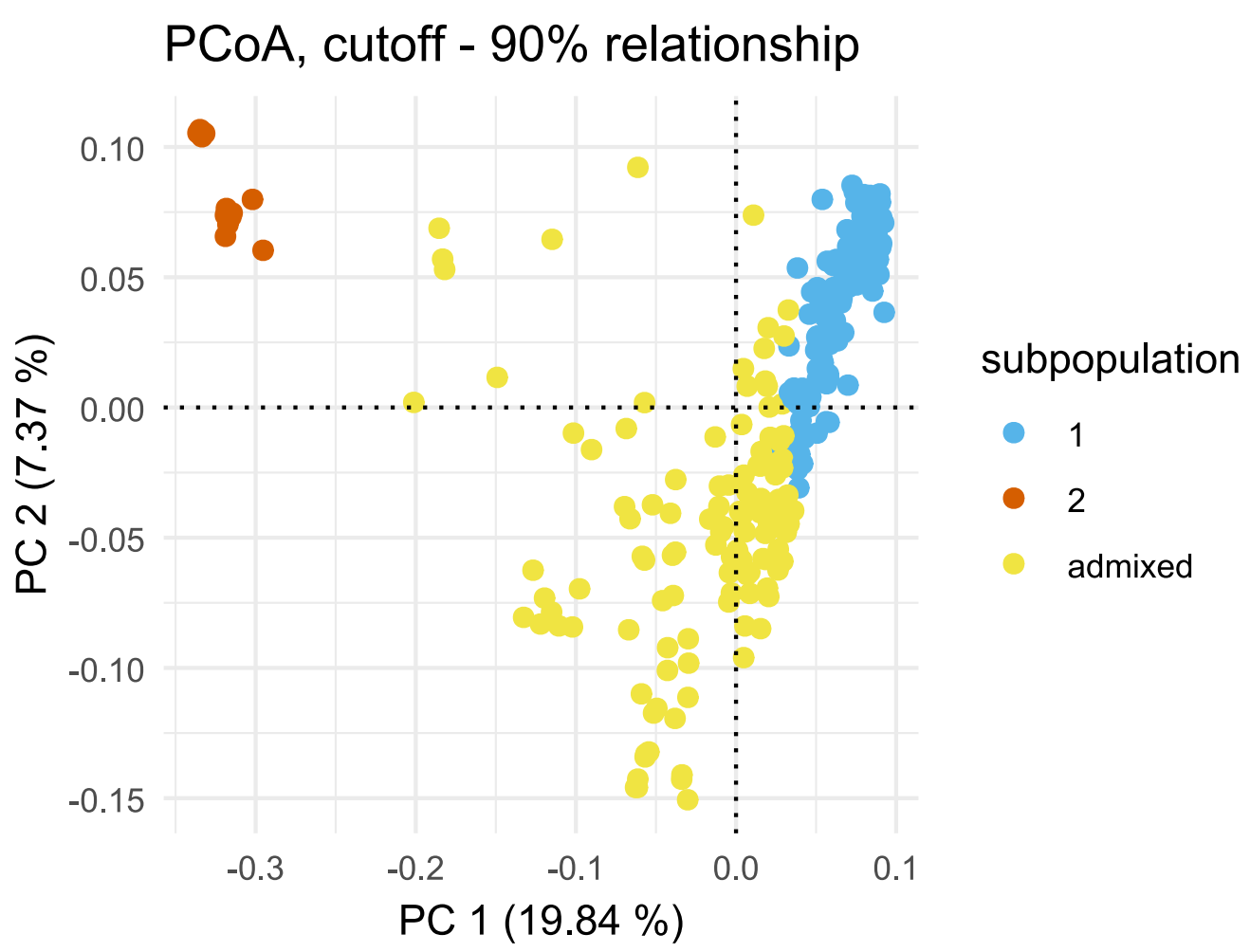

PCoA, cutoff - 90% relationship
0.10
0.05
0.00
-0.05
-0.10
-0.15
PC 2 (7.37 %)
-0.3
-0.2
-0.1
0.0
0.1
PC 1 (19.84 %)
subpopulation
1
2
admixed
