# Supplementary material for: Modern pea (Lathyrus oleraceus) cultivars exhibit significantly lower genetic diversity relative to landraces, garden cultivars and genebank accessions
Source: PLoS One. 2026 Oct 21;21(10):e0357113. doi: 10.1371/journal.pone.0357113 (PMC13686274; doi:10.1371/journal.pone.0357113)
Supplement: Supplementary file 3 [file pone.0357113.s005.pdf]

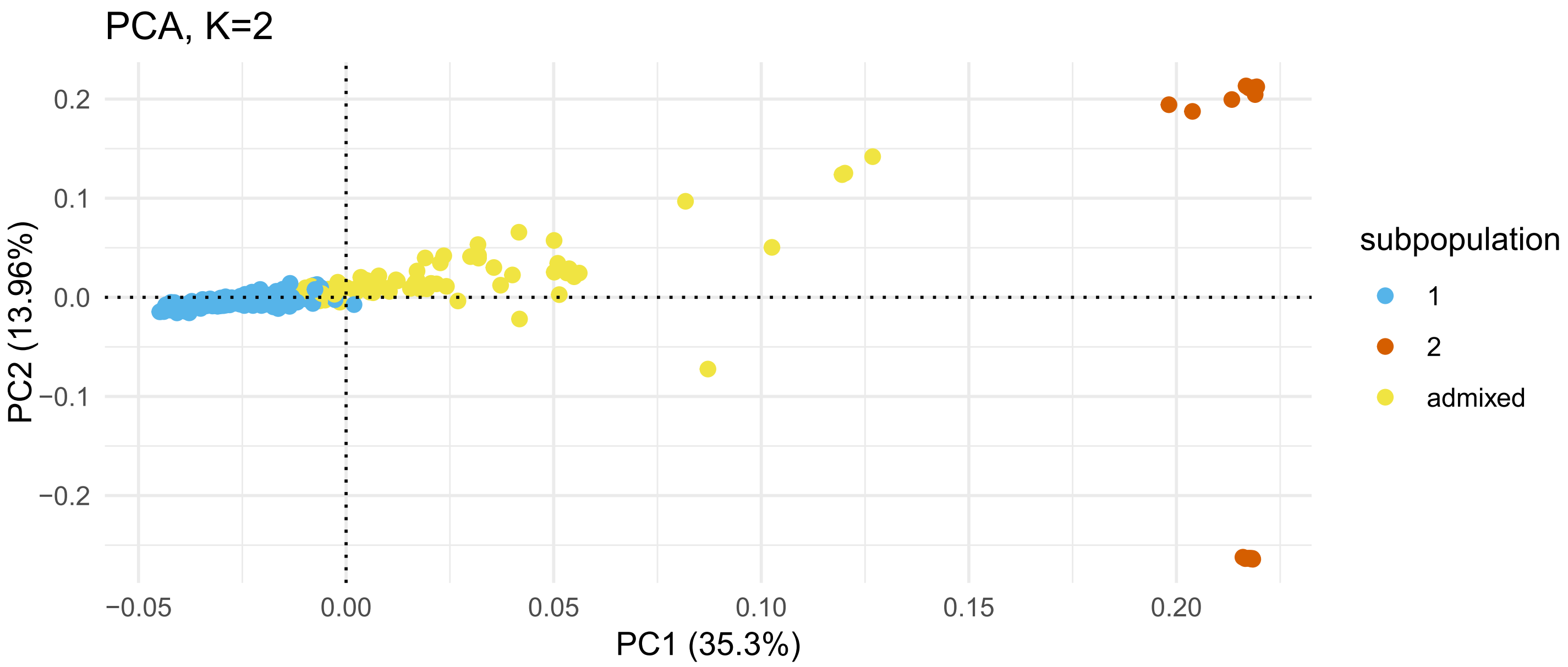

PCA, K=2
0.2
0.1
0.0
−0.1
−0.2
PC2 (13.96%)
−0.05
0.00
0.05
0.10
0.15
0.20
PC1 (35.3%)
subpopulation
1
2
admixed

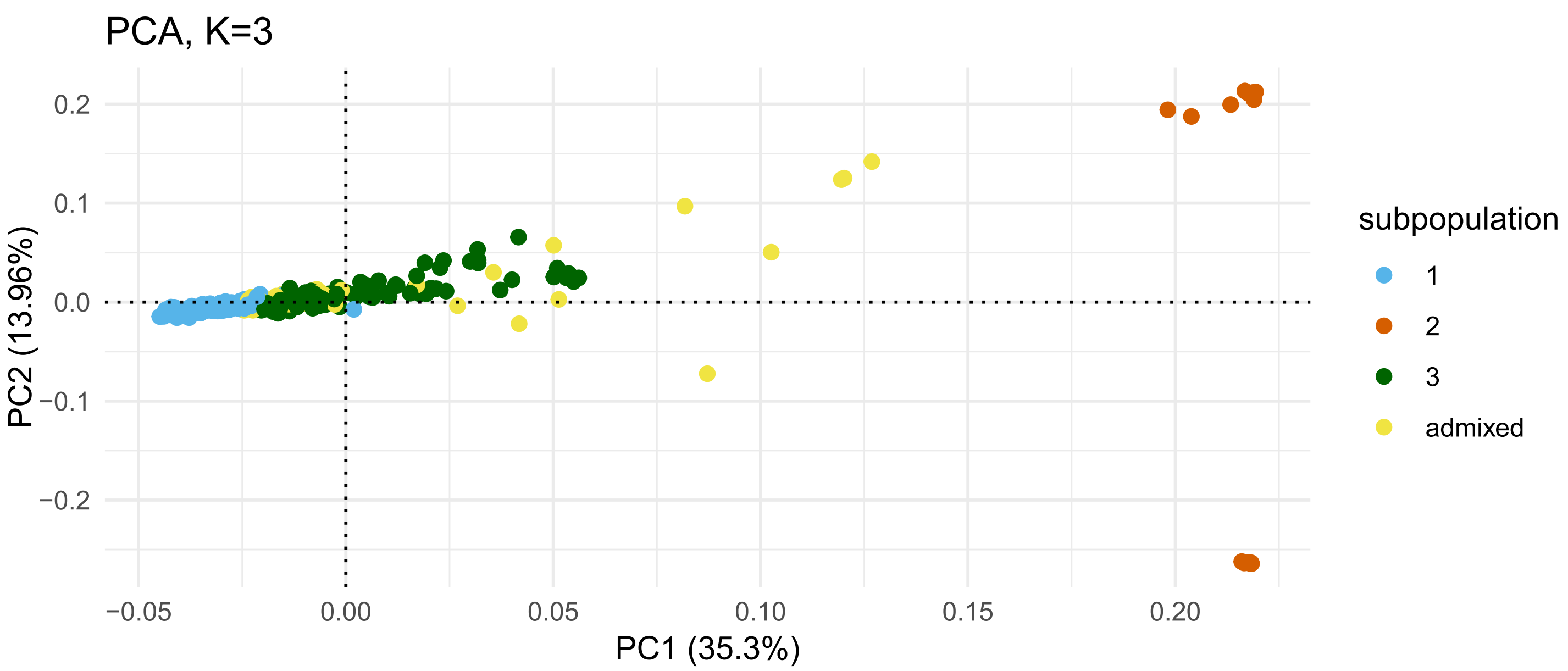

PCA, K=3
0.2
0.1
0.0
−0.1
−0.2
PC2 (13.96%)
−0.05
0.00
0.05
0.10
0.15
0.20
PC1 (35.3%)
subpopulation
1
2
3
admixed
