# Supplementary material for: Modern pea (Lathyrus oleraceus) cultivars exhibit significantly lower genetic diversity relative to landraces, garden cultivars and genebank accessions
Source: PLoS One. 2026 Oct 21;21(10):e0357113. doi: 10.1371/journal.pone.0357113 (PMC13686274; doi:10.1371/journal.pone.0357113)
Supplement: Supplementary file 4 [file pone.0357113.s006.docx]

| Population | Ho | He | Fis |
| --- | --- | --- | --- |
| Global population | 0.014 | 0.251 | 0.944 |
| 1 (modern cultivars, landraces and more) | 0.014 | 0.255 | 0.945 |
| 2 (Abyssinicum and Indian and Nepalese Landraces) | 0.011 | 0.161 | 0.932 |
| Admixed group | 0.017 | 0.330 | 0.948 |
