# Supplementary material for: Modern pea (Lathyrus oleraceus) cultivars exhibit significantly lower genetic diversity relative to landraces, garden cultivars and genebank accessions
Source: PLoS One. 2026 Oct 21;21(10):e0357113. doi: 10.1371/journal.pone.0357113 (PMC13686274; doi:10.1371/journal.pone.0357113)
Supplement: Supplementary file 6 [file pone.0357113.s007.docx]

| Population | 1 | 2 |
| --- | --- | --- |
| 1 |  |  |
| 2 | 0.497 |  |
| Admixed | 0.099 | 0.366 |
